# A clinicopathological analysis of forkhead box A1 (FOXA1) and estrogen receptor alpha expression in extramammary Paget’s disease

**DOI:** 10.20407/fmj.2022-030

**Published:** 2023-05-09

**Authors:** Chiho Sumitomo, Yohei Iwata, Masaru Arima, Kazumitsu Sugiura

**Affiliations:** Department of Dermatology, Fujita Health University, School of Medicine, Toyoake, Aichi, Japan

**Keywords:** EMPD, ER, Estrogen receptor, Extramammary Paget’s disease, FOXA1

## Abstract

**Objectives:**

Extramammary Paget’s disease (EMPD) is a neoplastic skin disease of unknown etiology. EMPD is frequently associated with forkhead box A1 (FOXA1) expression, which correlates with the expression of estrogen receptor alpha (ER). FOXA1 regulates the transcriptional activity of ER and may function cooperatively in the tumorigenesis of breast cancer.

**Methods:**

We performed immunohistochemical staining for FOXA1 and ER using tissue samples from 16 patients with EMPD.

**Results:**

The nuclei of Paget cells isolated from each of the 16 patients with EMPD (100%) were strongly FOXA1-positive, and the FOXA1 staining intensity was similar across all samples. ER staining was detected in the nuclei of Paget cells originating from seven patients with EMPD (44%), and the ER staining intensity varied between these patients.

**Conclusions:**

In the present study, we confirmed that EMPD was frequently associated with FOXA1 expression. However, ER expression varied between patients and did not always coincide with FOXA1 expression. No clear relationship was observed between ER expression, the intensity of ER staining, or EMPD metastasis and prognosis. However, the results indicate that hormone-dependent cancer therapy may be effective in patients with ER-positive EMPD.

## Introduction

Paget’s disease, which was first reported by Sir James Paget in 1874, can be of mammary or extramammary nature.^1^ Mammary Paget’s disease (MPD) is a rare form of breast cancer, which accounts for only 1%–4.3% of all breast cancers and is often associated with intraglandular, non-invasive, or invasive neoplasms.^1^ This rare disease is more commonly found in postmenopausal women, particularly after the age of 60 years; however, MPD has also been reported in adolescent women and in men.^1^ Extramammary Paget’s disease (EMPD) was first reported by Radcliffe Crocker in 1989 in a male patient with bladder cancer, who had eczema-like lesions on the penis and scrotum.^1^ EMPD shares several clinicopathological traits with MPD but differs in its pathogenesis.^2^ The cause of EMPD remains unknown.^3^ EMPD is classified as either primary or secondary, based on the absence or presence of associated malignancies. Primary EMPD is a disease of intraepithelial tissue, whereas, secondary EMPD is characterized by localized or metastasized cancerous tumors.^1^ In cases of resectable EMPD, surgery is recommended. However, unresectable EMPD is treated with chemotherapy, which is ineffective and is associated with a poor prognosis. For patients with systemic metastases, including extensive lymph node metastases, systemic chemotherapy does not improve the overall survival time.^4^ Moreover, as most patients with EMPD are older adults, the options for systemic chemotherapy are limited.^4^ Thus, tumor invasion levels and the presence or absence of multiple lymph node metastases are important prognostic factors for EMPD.^4^

The forkhead box A1 (FOXA1)- and nuclear receptor (NR)-regulated transcriptional programs are tightly coupled in breast cancer.^5^ FOXA1 regulates the ability of steroid NRs to control transcription, predominantly in hormone-responsive tissues such as the breast. Thus, the FOXA1/NR axis plays a critical role in both organ development and cancer progression.^6^ NRs such as estrogen, progesterone, and androgen receptors are abundantly expressed in the majority of breast cancers, where they serve as critical regulators of tumor growth and metastasis.^5^ Among the ~40 NR family members expressed in breast cancer, the estrogen receptor alpha (ER) isoform is the most clinically associated with FOXA1.^5^

EMPD is frequently linked to FOXA1 expression.^3^ Therefore, we performed immunostaining for FOXA1 and ER using skin tissue samples from 16 patients diagnosed with EMPD at our hospital between January 2019 and December 2021.

## Methods

To determine the association between EMPD and FOXA1, we examined the expression of FOXA1 in Paget cells isolated from patients with EMPD. Because FOXA1 regulates the transcriptional activity of ER, immunohistochemical (IHC) staining for ER was also performed. Skin specimens were retrieved from the archives, originating from 16 patients who were diagnosed with primary EMPD (based on IHC results) and underwent total resection at the Fujita Health University between January 2019 and December 2021. In total, we included eight men and eight women; mean age±standard deviation, 72±10 years; range, 51–93 years. The specimens were then prepared for hematoxylin and eosin (H&E), FOXA1, and ER staining. IHC staining for FOXA1 and ER was performed on paraffin sections using an HX System Discovery Automated Immunostainer (Roche, Basel, Switzerland) with a polymer-based detection system, according to the manufacturer’s instructions. FOXA1 IHC staining was performed using an anti-FOXA1 antibody (clone [EPR10881], ab170933, Abcam, Cambridge, UK), a rabbit monoclonal antibody targeting the C-terminal amino acids of the FOXA1 protein. IHC staining with a primary anti- estrogen receptor alpha antibody (clone [SP1], ab16660, Abcam) was performed according to the manufacturer’s instructions. 3,3'-Diaminobenzidine (DAB) was used as a color-developing reagent in IHC assays. The pattern of ER staining was assessed according to the Allred score^7^ and the samples were categorized into two groups: + (Allred score ≥3) or – (Allred score <3). The staining pattern of FOXA1 was evaluated in terms of staining rate and intensity. All IHC analyses were performed by independent researchers. Written informed consent was obtained from all patients and tissue donors, in accordance with the Declaration of Helsinki. Informed consent for skin sample collection was obtained from each participant according to the protocols approved by the Institutional Review Board of Fujita Health University (HM19-450).

## Results

**Figure 1:**
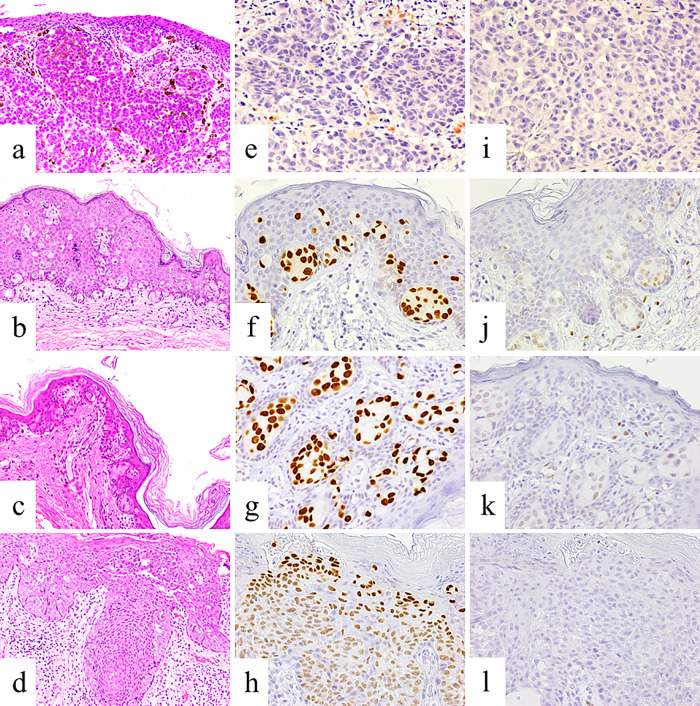
Immunohistochemical analyses of extramammary Paget’s disease and melanoma patient samples. Control: a 73-year-old man with melanoma of the upper right arm. (a) Hematoxylin and eosin (H&E) staining. Tumor cells were negative for forkhead box A1 (FOXA1) (e) and estrogen receptor (ER)α (i). Patient 6: (b) H&E staining. Tumor cells were positive for FOXA1 (f) and ERα (j). Patient 10: (c) H&E staining. Tumor cells were positive for FOXA1 (g) and ERα (k). Patient 16: (d) H&E staining. Tumor cells were positive for FOXA1 (h) but negative for ERα (l). a–d. 200× magnification; e–l. 400× magnification.

The histopathological features of the 16 patients with EMPD are summarized in Table 1. FOXA1 was strongly expressed in the nuclei of Paget cells from all examined patients with EMPD. Moreover, the FOXA1 staining intensity was similar in all the samples. In contrast, FOXA1^+^ cells were almost completely absent from the five age-matched melanoma skin samples (obtained from two men and three women; mean age±standard deviation, 73±14 years; range, 54–92 years) (Figure 1). Alkaline phosphatase (AP) was used as a color-developing reagent for FOXA1 and ER IHC staining of melanoma samples. According to the Allred score, 7 (44%) of the 16 patients with EMPD were ER^+^, with varying staining intensities. There was no association between age or sex and ER^+^ staining; the ER^+^ patients 3, 6, 9, 10, 11, 13, and 14 were three men and four women; mean age±standard deviation, 72±12 years; range, 51–93 years. The primary locations of ER-positive cases were vulval (all four women) and scrotal (all three men). A 77-year-old woman (patient 3) had a metastasis in the sentinel lymph node (SLN). Two (29%) of the six ER-positive patients had invasive EMPD, while, 6 (35%) of the 16 patients with EMPD had invasive disease. In addition, four patients (patients 6, 9, 10, and 14; three men and one woman; mean age±standard deviation, 76±11 years; range, 68–93 years) had high ER staining, which was the highest in samples from male patients. Of the four ER^Hi^ patients, one patient (25%) had severe dermal invasion; no cases of SLN metastasis were observed.

## Discussion

NRs such as estrogen, progesterone, and androgen receptors are abundantly expressed in the majority of breast cancers, where they serve as critical regulators of tumor growth and metastasis.^5^ FOXA1 is also highly expressed in breast cancer, and its binding to DNA helps define which genes are regulated by the NRs.^5^ FOXA1 establishes estrogen-responsive transcriptomes in luminal breast cancer.^5^ Dysregulated FOXA1 chromatin occupancy through focal amplification, mutation, or cofactor recruitment modulates ER transcriptional programs and drives endocrine-resistant disease.^5^ However, ER is not the sole NR expressed in breast cancers, nor is it the only NR for which FOXA1 serves as a licensing factor.^5^ Receptors for androgens, glucocorticoids, and progesterone are also implicated in many breast cancers, and their functions are also regulated by FOXA1.^5^ These NRs interfere with ER transcriptional programs and, depending on their activation level, can reprogram FOXA1-ER cistromes.^5^ Thus, interplay between NRs contributes to the endocrine response to therapy and drug resistance, which may help develop future therapeutic strategies for patients with hormone-dependent cancer.^5^

Among the ~40 NR family members expressed in breast cancer, ER is the most clinically associated with FOXA1.^5^ A second isoform of the estrogen receptor, ERβ, is also expressed in breast cancer; however, the relative contribution of ERβ in this disease is poorly understood.^8^

The expression of ER in breast cancer significantly dictates the treatment course and is a major prognostic factor.^5^ Indeed, the therapeutic targeting of ER signaling using selective ER modulators (e.g., aromatase inhibitors) or degraders provides long-term benefits for the majority of patients with early stage disease and extends the lives of many patients with advanced lesions.^5^

FOXA1 is known to support the transcriptional activity of ER.^9^ Takeichi et al. reported the results of FOXA1 and ER IHC staining using samples from 48 patients with EMPD.^3^ They showed that FOXA1 was strongly expressed in the patients’ Paget cell nuclei at the same staining intensity in all patients, while 65% of the patients exhibited positive ER staining, which varied in intensity across the patient cohort. In agreement with these results, we found that FOXA1 was strongly expressed in the nuclei of Paget cells in all examined patients with EMPD, and that this staining intensity was consistent across all samples. In the present study, the rate of positive ER staining was lower (44%). However, Mai et al. previously reported that 88% of the 59 patients with EMPD were FOXA1-positive and 19% were ER-positive.^10^ The authors also found that in patients with EMPD, ER expression always coincided with FOXA1 expression, which is consistent with the results of our study.^10^ FOXA1 overexpression suppresses interferon signaling and the anti-tumor immune response. Thus, FOXA1 overexpression could be a prognostic factor for predicting resistance to anti-cancer therapy.^11^ We were able to reconfirm that not all FOXA1-positive patients were ER-positive. These findings suggest that in a subset of FOXA1^+^ER^–^ patients, FOXA1 is activated by mechanisms other than ER. However, since FOXA1 is a novel diagnostic marker for EMPD, ER may aid in the molecular classification of EMPD. Thus, FOXA1 and ER may be valuable molecular targets for the development of EMPD therapies.

The therapeutic targeting of ER signaling is an effective strategy for breast cancer management. Our results suggest that certain treatment options for hormone-dependent cancers may also be effective at treating patients with ER-positive EMPD. In our study, ER expression was localized to the vulva and scrotum of women and men with EMPD, respectively. Therefore, detection of ER expression in these tissues may aid the diagnosis and treatment of patients with EMPD. However, there are currently no studies documenting the site of onset or the clinical relevance of ER-positive EMPD. The present study was limited by the small sample size, which should be increased in future investigations. In addition, the relationship between EMPD and other NRs such as the human epidermal growth factor receptor 2 (HER2) should be examined in the future.

## Figures and Tables

**Table1:** The histopathological features of patients with EMPD

Patient number	Age/sex	Age at onset	Primary location	Dermal invasion	Metastasis of SLN^a^	FOXA1 expression	ERα expression
1	80/M	80	Inguinal	invasive	+	+	−
2	68/M	67	Penoscrotal	invasive	−	+	−
3	77/F	77	Vulval	invasive	+	+	+
4	78/M	77	Penoscrotal	invasive	+	+	−
5	72/F	71	Vulval	in situ	−	+	−
6	72/M	72	Scrotal	in situ	−	+	+
7	67/F	61	Axillary	in situ	−	+	−
8	90/M	90	Axillary	in situ	−	+	−
9	72/F	68	Vulval	invasive	−	+	+
10	95/M	93	Scrotal	in situ	−	+	+
11	71/F	70	Vulval	in situ	−	+	+
12	68/F	65	Vulval	invasive	−	+	−
13	55/F	51	Vulval	in situ	−	+	+
14	71/M	71	Scrotal	in situ	−	+	+
15	72/F	69	Vulval	in situ	−	+	–
16	69/M	68	Perianal	in situ	−	+	−

^a^ SLN, sentinel lymph node.

## References

[1] Lopes Filho LL, Lopes IM, Lopes LR, Enokihara MMSS, Michalany AO, Matsunaga N. Mannary and extramammary paget’s disease. An Bras Dermatol 2015; 90: 225–231. 25830993 10.1590/abd1806-4841.20153189 PMC4371672

[2] Kanitakis J. Mammary and extramammary Paget’s disease. J Eur Acad Dermatol Venereol 2007; 21: 581–590. 17447970 10.1111/j.1468-3083.2007.02154.x

[3] Takeichi T, Okuno Y, Matsumoto T, Tsunoda N, Suzuki K, Takahashi K, Kono M, Kikumori T, Muto Y, Akiyama M. Frequent FOXA1-activation mutations in extramammary Paget’s disease. Cancers 2020; 12: 820. 32235312 10.3390/cancers12040820 PMC7226542

[4] Hatta N, Yamada M, Hirano T, Fujimoto A, Morita R. Extramammary Paget’s disease: treatment, prognostic factors and outcome in 76 patients. Br J Dermatol 2008; 158: 313–318. 18028492 10.1111/j.1365-2133.2007.08314.x

[5] Seachrist DD, Anstine LJ, Keri RA. FOXA1: a pioneer of nuclear receptor action in breast cancer. Cancers 2021; 13: 5205. 34680352 10.3390/cancers13205205 PMC8533709

[6] Bernardo GM, Keri RA. FOXA1: A transcription factor with parallel functions in development and cancer. Biosci Rep 2012; 32: 113–130. 22115363 10.1042/BSR20110046 PMC7025859

[7] Allred DC, Harvey JM, Berardo M, Clark GM. Prognostic and predictive factors in breast cancer by immunohistochemical analysis. Mod Pathol 1998; 11: 155–168. 9504686

[8] Haldosén LA, Zhao C, Dahlman-Wright K. Estrogen receptor beta in breast cancer. Mol Cell Endocrinol 2014; 382: 665–672. 23954741 10.1016/j.mce.2013.08.005

[9] Zhang Y, Zhang D, Li Q, et al. Nucleation of DNA repair factors by FOXA1 Links DNA demethylation to transcriptional pioneering. Nat Genet 2016; 48: 1003–1013. 27500525 10.1038/ng.3635

[10] Mai R, Zhou S, Zhou S, et al. Transcriptome analyses reveal FOXA1 dysregulation in mammary and extramammary Paget’s disease. Hum Pathol 2018; 77: 152–158. 29630912 10.1016/j.humpath.2017.12.030

[11] He Y, Wang L, Wei T, et al. FOXA1 overexpression suppresses interferon signaling and immune response in cancer. J Clin Invest 2021; 131: e147025. 34101624 10.1172/JCI147025 PMC8279591

